# Identification and evolution of glucosinolate sulfatases in a specialist flea beetle

**DOI:** 10.1038/s41598-019-51749-x

**Published:** 2019-10-31

**Authors:** Seung-Joon Ahn, Franziska Betzin, Matilda W. Gikonyo, Zhi-Ling Yang, Tobias G. Köllner, Franziska Beran

**Affiliations:** 1 Research Group Sequestration and Detoxification in Insects, Max Planck Institute for Chemical Ecology, Hans-Knöll-Str. 8, 07745 Jena, Germany; 2 Department of Biochemistry, Max Planck Institute for Chemical Ecology, Hans-Knöll-Str. 8, 07745 Jena, Germany; 3 Present Address: Department of Biochemistry, Molecular Biology, Entomology and Plant Pathology, Mississippi State University, Mississippi State, Mississippi 39762 United States

**Keywords:** Ecophysiology, Molecular evolution

## Abstract

Glucosinolates, a characteristic group of specialized metabolites found in Brassicales plants, are converted to toxic isothiocyanates upon herbivory. Several insect herbivores, including the cabbage stem flea beetle (*Psylliodes chrysocephala*), prevent glucosinolate activation by forming desulfo-glucosinolates. Here we investigated the molecular basis of glucosinolate desulfation in *P. chrysocephala*, an important pest of oilseed rape. Enzyme activity assays with crude beetle protein extracts revealed that glucosinolate sulfatase (GSS) activity is associated with the gut membrane and has narrow substrate specificity towards the benzenic glucosinolate sinalbin. In agreement with GSS activity localization *in vivo*, we identified six genes encoding arylsulfatase-like enzymes with a predicted C-terminal transmembrane domain, of which five showed GSS activity upon heterologous expression in insect cells. *Pc*GSS1 and *Pc*GSS2 used sinalbin and indol-3-ylmethyl glucosinolate as substrates, respectively, whereas *Pc*GSS3, *Pc*GSS4, and *Pc*GSS5 showed weak activity in enzyme assays. RNAi-mediated knock-down of *PcGSS1* and *PcGSS2* expression in adult beetles confirmed their function *in vivo*. In a phylogenetic analysis of coleopteran and lepidopteran arylsulfatases, the *P. chrysocephala* GSSs formed a cluster within a coleopteran-specific sulfatase clade distant from the previously identified GSSs of the diamondback moth, *Plutella xylostella*, suggesting an independent evolution of GSS activity in ermine moths and flea beetles.

## Introduction

Herbivorous insects encounter a variety of chemical defense metabolites in their plant diet and thus require efficient strategies to prevent intoxication^1,2^. Many plant defense metabolites are stored as non-toxic glucosides (pro-toxins) and are hydrolyzed to reactive aglucones in the herbivore gut by plant or insect *β*-glucosidases. Insect adaptations to such activated plant defenses include detoxification or re-glycosylation of the reactive aglucones, inhibition of *β*-glucosidase activity, and enzymatic detoxification of the pro-toxin^3–7^.

The glucosinolate-myrosinase system is a well-studied activated defense in plants of the order Brassicales, which includes important crops such as cabbage, mustard, and radish^8^. Glucosinolates are *β*-D-thioglucoside-*N*-hydroxysulfates derived from different amino acids such as Trp (indolic glucosinolates), Phe (benzenic glucosinolates), Met and other aliphatic amino acids (aliphatic glucosinolates)^8,9^. Upon tissue damage, glucosinolates are rapidly hydrolyzed by plant *β*-thioglucosidase enzymes (myrosinases) to unstable aglucones that spontaneously degrade to reactive isothiocyanates and other breakdown products, which are responsible for the characteristic flavor and smell of crucifer vegetables^10^. Glucosinolate breakdown products are deterrent and toxic to non-adapted small herbivores and microorganisms, whereas adapted species can either prevent glucosinolate breakdown to toxic isothiocyanates, or detoxify isothiocyanates by conjugation to glutathione^11,12^.

The cabbage stem flea beetle, *Psylliodes chrysocephala*, a serious pest of winter oilseed rape in Europe^13^, is specialized to feed on glucosinolate-containing plants^14^. A previous study revealed that *P. chrysocephala* selectively sequesters glucosinolates, and additionally detoxifies glucosinolates by conversion to desulfo-glucosinolates, which are not a substrate of myrosinase^15^. These two strategies decrease the fraction of ingested glucosinolates that are otherwise activated to toxic breakdown products. According to a quantitative analysis of 4-methylsulfinylbutyl glucosinolate metabolism in *P. chrysocephala*, about 8% of the total ingested glucosinolates were detoxified by desulfation, indicating small but significant glucosinolate sulfatase (GSS) activity towards 4-methylsulfinylbutyl glucosinolates in adult beetles^15^.

The ability to detoxify glucosinolates by desulfation was first discovered in the diamondback moth, *Plutella xylostella*. In this specialist, GSS activity is localized in the larval gut lumen where most ingested glucosinolates are rapidly converted to desulfo-glucosinolates and then excreted^16,17^. *P. xylostella* possesses three GSS enzymes, which differ in their substrate specificities and expression patterns. While *Px*GSS1 desulfates all classes of glucosinolates, *Px*GSS2 and *Px*GSS3 only detoxify long-chain Met-derived glucosinolates, and Phe- and Trp-derived glucosinolates, respectively. In agreement with their substrate spectra, *PxGSS1* and *PxGSS2* are inducible by Met-derived glucosinolates, whereas Trp-derived glucosinolates induce *PxGSS3* expression. *PxGSS* genes are located in tandem in a conserved lepidopteran arylsulfatase gene cluster comprising *SulfB*, *SulfC*, and *SulfD*, and evolved from *SulfC* by gene duplications^17^. GSS activity is also present in the generalist desert locust, *Schistocerca gregaria*^18^, and in the phloem-feeding whitefly, *Bemisia tabaci*^19^, but the corresponding GSS enzymes are unknown. Moreover, *Athalia rosae* sawfly larvae metabolize sequestered glucosinolates to stable desulfo-glucosinolate-3-sulfates, which suggests that a glucosinolate sulfatase as well as a sulfotransferase are involved in glucosinolate metabolism in this specialist, although no GSS activity was detectable in larvae^20,21^. GSS activity assays performed with crude protein extracts of the flea beetle *Phyllotreta striolata* also did not reveal GSS activity^22^.

Here, we investigated the ability of the flea beetle *P. chrysocephala* to detoxify glucosinolates by desulfation. We demonstrate a gut-specific GSS activity in adults that is associated with the membrane fraction. In contrast to the broad GSS activity previously observed in *P. xylostella* and *S. gregaria*, gut protein extracts of *P. chrysocephala* were primarily active towards the uncommon benzenic glucosinolate *p*-hydroxybenzyl glucosinolate (sinalbin). Based on homology to known insect arylsulfatases, we identified six genes in a *P. chrysocephala* transcriptome that encode arylsulfatase-like enzymes with a C-terminal transmembrane domain. Functional characterization of recombinant enzymes and RNAi studies revealed that *P. chrysocephala* possesses at least two GSS enzymes, *Pc*GSS1 and *Pc*GSS2, which detoxify sinalbin and indol-3-ylmethyl glucosinolate, respectively. The function of three additional arylsulfatase-like enzymes with minimal GSS activity under our assay conditions remains unknown. Finally, we explored the evolution of GSS activity in Coleoptera and Lepidoptera in a phylogenetic analysis.

## Results

### Localization of glucosinolate sulfatase activity in *P. chrysocephala*

**Figure 1:**
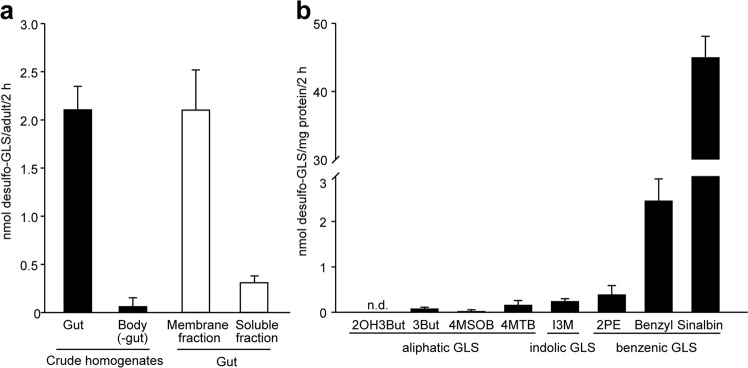
Glucosinolate sulfatase (GSS) activity in *Psylliodes chrysocephala* adults. (**a**) Crude tissue homogenates were prepared from dissected guts and the remaining body tissues, and gut tissue homogenates were fractionated into membrane fraction and soluble protein fraction by centrifugation. Samples were incubated with a mixture of eight different glucosinolates (GLS) for 2 h at 35 °C, and desulfo-glucosinolates were quantified by LC-MS/MS using external standard curves. (**b**) Comparison of GSS activity in crude gut homogenates towards eight different glucosinolates. Means + SD of n = 4 biological replicates. n.d., not detected; 2OH3But, 2-hydroxy-3-butenyl; 3But, 3-butenyl; 4MSOB, 4-methylsulfinylbutyl; 4MTB, 4-methylthiobutyl; I3M, indol-3-ylmethyl; 2PE, 2-phenylethyl.

To determine the localization of GSS activity in *P. chrysocephala* adults, we incubated crude tissue homogenates of dissected guts and the corresponding remaining body tissues with different glucosinolate substrates. These assays revealed 35-fold higher total GSS activity in the gut compared to the body (without gut). Further fractionation of the *P. chrysocephala* gut homogenate into soluble protein and cell membrane fractions showed that enzyme activity was mainly associated with the gut membrane (Fig. 1a). Of eight glucosinolates tested in enzyme assays, strong GSS activity was detected towards sinalbin, whereas the activity towards all other glucosinolates tested was below 5% of that towards sinalbin (Fig. 1b). In contrast, enzyme activity assays performed with gut tissue homogenates of the horseradish flea beetle, *Phyllotreta armoraciae,* revealed no GSS activity (Supplementary Fig. S1).

### Identification and functional characterization of putative arylsulfatases from *P. chrysocephala*

Based on amino acid sequence similarity to *Px*GSS1, we identified nine arylsulfatase-like genes in a *P. chrysocephala* transcriptome, and obtained their full-length open reading frames by rapid amplification of cDNA ends PCR (GenBank accession numbers KX986114 to KX986122; Table 1, Supplementary Data S1). The encoded proteins share between 34% and 93% amino acid sequence identity, and between 30% and 34% sequence identity with *Px*GSS1 from *P. xylostella*. In eukaryotic arylsulfatases, the catalytic Cα-formylglycine (FGly) residue is formed via post-translational modification of a conserved cysteine residue in sulfatase signature sequence I. This cysteine residue is present in all *P. chrysocephala* arylsulfatases except for *Pc*Sulf2, which contains a serine-residue in this position (Supplementary Fig. S2). Serine-type arylsulfatases are known in prokaryotes, but so far not in eukaryotes^23,24^. The second sulfatase signature sequence contains conserved lysine and histidine residues involved in sulfate ester cleavage. In *Pc*GSS3, *Pc*GSS4, and *Pc*GSS5, the conserved histidine residue is substituted with an asparagine residue, which likely affects their catalytic activity. All arylsulfatase-like genes encode proteins with a predicted N-terminal secretion signal, predicting an extracellular localization. A predicted C-terminal transmembrane domain in six arylsulfatase-like enzymes further suggests that these are membrane-bound (Supplementary Fig. S2, Table 1).

**Table 1:** Properties of predicted arylsulfatase-like enzymes identified in *Psylliodes chrysocephala* and two *Phyllotreta* species.

Species	Gene name	GenBank accession no.	Length, aa	Molecular mass, kDa^a^	Isoelectric point^a^	Predicted *N*-glycosylation sites	Signal peptide, aa	Transmembrane domain, aa
*Psylliodes chrysocephala*	*PcSulf1*	KX986114	554	59.56	7.06	7	21	—
*Psylliodes chrysocephala*	*PcSulf2*	KX986115	559	59.77	5.03	1	22	—
*Psylliodes chrysocephala*	*PcSulf3*	KX986116	583	63.81	7.68	6	18	—
*Psylliodes chrysocephala*	*PcSulf4*	KX986117	620	68.64	7.41	4	19	577–598
*Psylliodes chrysocephala*	*PcGSS1*	KX986118	623	69.20	6.67	4	19	579–599
*Psylliodes chrysocephala*	*PcGSS2*	KX986119	634	70.47	6.11	5	19	587–608
*Psylliodes chrysocephala*	*PcGSS3*	KX986120	614	68.17	8.49	3	19	570–589
*Psylliodes chrysocephala*	*PcGSS4*	KX986121	614	68.41	8.88	3	19	570–589
*Psylliodes chrysocephala*	*PcGSS5*	KX986122	614	68.15	8.87	3	19	567–589
*Phyllotreta striolata*	*PsSulf1*	KX986123	551	58.68	6.48	4	24	—
*Phyllotreta striolata*	*PsSulf2*	KX986124	556	59.84	5.07	1	20	—
*Phyllotreta striolata*	*PsSulf3*	KX986125	585	63.62	6.50	7	21	—
*Phyllotreta striolata*	*PsSulf4*	KX986126	617	67.74	6.34	3	17	574–595
*Phyllotreta armoraciae*	*PaSulf1*	KX986127	551	59.07	6.88	5	22	—
*Phyllotreta armoraciae*	*PaSulf2*	KX986128	556	59.69	5.11	1	20	—
*Phyllotreta armoraciae*	*PaSulf3*	KX986129	585	64.30	6.16	8	20	—
*Phyllotreta armoraciae*	*PaSulf4*	KX986130	618	67.94	6.23	4	17	574–595

^a^The molecular mass and isoelectric point of each protein was computed by ProtParam tool using the full-length amino acid sequence without the predicted signal peptide.

Next, we expressed these nine arylsulfatase-like genes in *Sf*9 insect cells to determine their enzymatic activity towards the general substrate 4-nitrocatechol sulfate and different glucosinolate substrates *in vitro*. To obtain soluble protein for activity assays, enzymes were expressed without the C-terminal transmembrane domain. The protein expression levels differed strongly, and several recombinant enzymes were only detected by Western blotting after 50-fold concentration of the culture medium (Supplementary Fig. S3).

In enzyme assays, only *Pc*Sulf4 showed arylsulfatase activity towards the general substrate 4-nitrocatechol sulfate (Supplementary Fig. S4), whereas five enzymes were active towards different glucosinolates (Table 2). Recombinant *Pc*GSS1 was active towards sinalbin and additionally showed minor activity towards benzyl glucosinolate (Table 2, Supplementary Fig. S5). Indol-3-ylmethyl glucosinolate was the major substrate of *Pc*GSS2, which also showed trace activities towards all benzenic glucosinolates and 4-methylsulfinylbutyl glucosinolate (Table 2, Supplementary Fig. S5). Recombinant *Pc*GSS3 and *Pc*GSS4 converted 3-butenyl glucosinolate, 4-methylthiobutyl glucosinolate, and 2-phenylethyl glucosinolate into the corresponding desulfo-glucosinolates, while *Pc*GSS5 showed only trace activity towards 2-phenylethyl glucosinolate (Table 2). Considering the widely differing protein expression levels, the enzymatic activities of recombinant *Pc*GSS3, *Pc*GSS4, and *Pc*GSS5 were much weaker than those of *Pc*GSS1 and *Pc*GSS2 under our assay conditions.

**Table 2:** Glucosinolate sulfatase activity of recombinant enzymes towards different glucosinolates (GLS).

GLS	pmol desulfo-GLS in 2 h (mean ± SD; n = 3)				
	*Pc*GSS1	*Pc*GSS2	*Pc*GSS3	*Pc*GSS4	*Pc*GSS5
2OH3But	n.d.	n.d.	n.d.	n.d.	n.d.
3But	n.d.	n.d.	4.18 ± 1.95	20.39 ± 3.88	n.d.
4MSOB	n.d.	3.04 ± 0.32	n.d.	n.d.	n.d.
4MTB	n.d.	n.d.	3.09 ± 0.43	0.48 ± 0.32	n.d.
I3M	n.d.	13,804.15 ± 582.38	n.d.	n.d.	n.d.
2PE	n.d.	1.73 ± 0.08	3.62 ± 2.42	6.55 ± 4.25	tr.
Benzyl	1.74 ± 0.94	13.47 ± 0.65	n.d.	n.d.	n.d.
Sinalbin	91.41 ± 40.18	6.60 ± 0.51	n.d.	n.d.	n.d.

2OH3But, 2-hydroxy-3-butenyl; 3But, 3-butenyl; 4MSOB, 4-methylsulfinylbutyl; 4MTB, 4-methylthiobutyl; I3M, indol-3-ylmethyl; 2PE, 2-phenylethyl; n.d., not detected; tr., trace amount.

**Figure 2:**
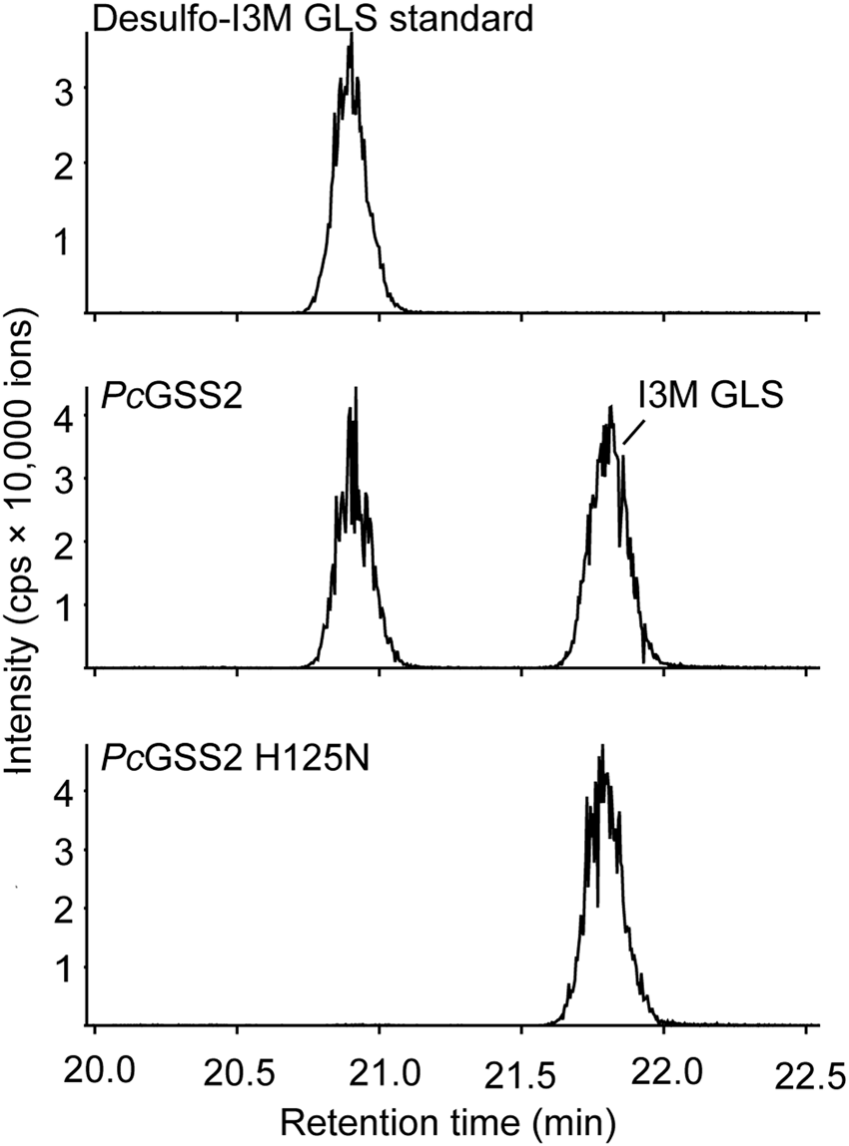
Mutation of the catalytic histidine residue at position 125 affects glucosinolate sulfatase (GSS) activity of *Pc*GSS2. *Pc*GSS2 and *Pc*GSS2 H125N were heterologously expressed in High Five^TM^ insect cells and incubated with eight different glucosinolate substrates for 2 h at 35 °C. Extracted LC-MS/MS ion chromatograms of desulfo-indol-3-ylmethyl glucosinolate (I3M glucosinolate) are shown. Remaining I3M glucosinolate in the assay was detected as desulfo-I3M glucosinolate due to in-source fragmentation in positive ionization mode.

To test whether the substitution of the catalytic histidine residue at position 125 by an asparagine residue is responsible for the low activities of *Pc*GSS3, *Pc*GSS4, and *Pc*GSS5, we generated the corresponding N125H mutants as well as the *Pc*GSS2 H125N mutant by site–directed mutagenesis. While mutation of the catalytic histidine residue in *Pc*GSS2 completely abolished activity towards indol-3-ylmethyl glucosinolate (Fig. 2), the substitution of asparagine with the catalytically active histidine residue did not affect the enzymatic activities of *Pc*GSS3 and *Pc*GSS4 towards the substrate 3-butenyl glucosinolate (Supplementary Fig. S6). Neither *Pc*GSS5 nor *Pc*GSS5 N125H showed GSS activity towards the tested glucosinolate substrates under our assay conditions (data not shown).

### Expression patterns of *PcSulf* and *PcGSS* genes

**Figure 3:**
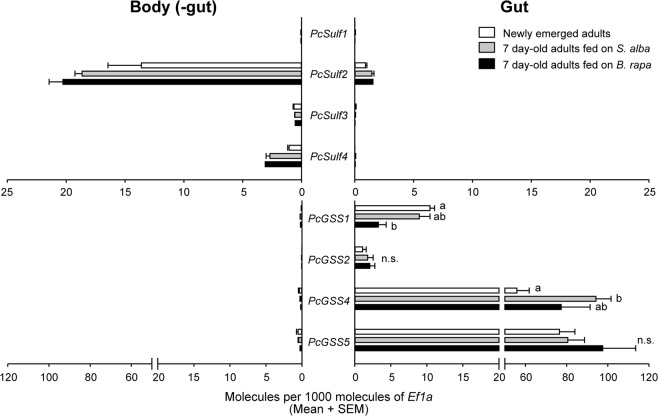
Expression patterns of *PcSulf* and *PcGSS* genes in *P. chrysocephala*. Gut tissue of newly emerged adults and adults, which had fed for seven days on *Sinapis alba* and *Brassica rapa*, respectively, was dissected. Total RNA extracted from homogenized guts and the corresponding bodies without guts was used for cDNA synthesis. The expression levels of *PcSulf* and *PcGSS* genes were determined relative to that of the reference gene *EF1α* by quantitative RT-PCR. *PcGSS3* expression was not analyzed because it was not possible to design gene-specific primers due to high sequence similarity among *PcGSS* genes. The expression level of each gene in the gut and the body without gut was compared by paired *t*-test (Table S1). GSS gene expression in the gut of newly emerged adults was compared with those in the guts of seven day-old adults which had fed on *S. alba* and *B. rapa*, respectively, by ANOVA followed by Tukey post-hoc test, or by Kruskal-Wallis test followed by Dunn’s post-hoc test (Table S2). Bars labeled with different letters are significantly different. Means + SE of n = 4–5 biological replicates.

The comparison of *PcSulf* and *PcGSS* transcript levels in the gut and remaining body tissues of *P. chrysocephala* by qRT-PCR showed that *PcSulf* genes were significantly higher expressed in the body (without gut), whereas *PcGSS* genes were significantly more expressed in the gut (Fig. 3; Supplementary Table S1). To assess whether glucosinolate ingestion affects *PcGSS* expression, we compared transcript levels in newly emerged adults and seven day-old adults fed on *Sinapis alba* (containing sinalbin as a major glucosinolate) or on *Brassica rapa* plants, respectively. *PcGSS1* transcript was less abundant in *B. rapa*-fed adults compared to newly emerged adults (decrease by 68%), whereas *PcGSS1* transcript levels in *S. alba*-fed adults did not differ significantly from those in newly emerged or *B. rapa*-fed adults. On the other hand, *PcGSS4* transcript was significantly more abundant in *S. alba*-fed adults than in newly emerged adults, but did not differ between *B. rapa*-fed beetles and the other two groups (Fig. 3, Supplementary Table S2). Interestingly, *PcGSS4* and *PcGSS5* expression levels were at least five times higher than those of *PcGSS1* and *PcGSS2* in *P. chrysocephala* (Fig. 3). *PcGSS3* expression was not analyzed due to high sequence similarity with other *PcGSS* genes.

### RNAi-mediated knock-down of *Pc*GSS1 and *Pc*GSS2 expression in *P. chrysocephala*

**Figure 4:**
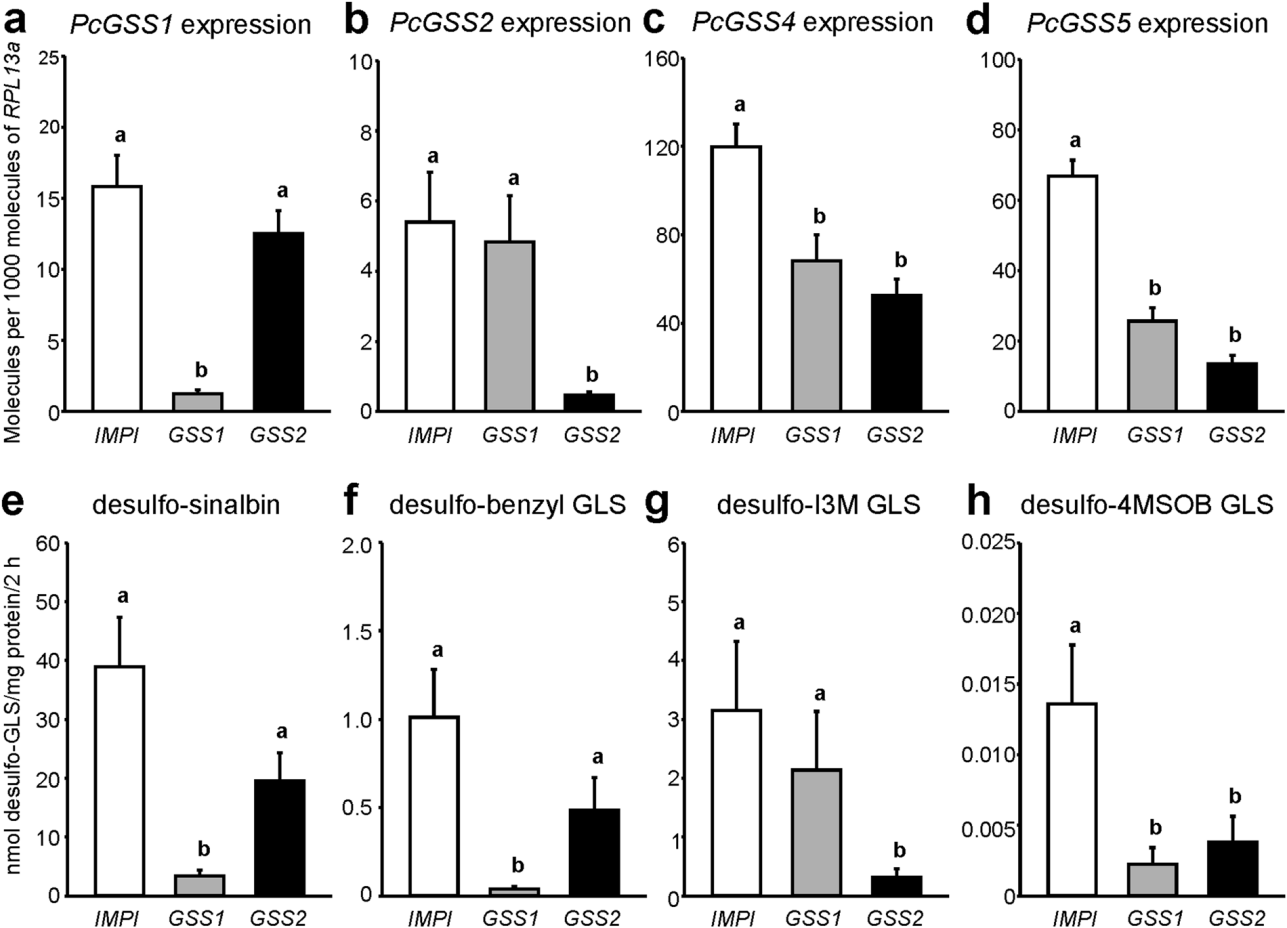
Knock-down of *PcGSS1* and *PcGSS2* gene expression in *P. chrysocephala* using RNA interference. Adults were injected with double-stranded RNA (dsRNA) targeting the 3′-UTR of *PcGSS1* (*GSS1*), *PcGSS2* (*GSS2*), and a lepidopteran-specific inducible metalloproteinase inhibitor gene from the greater wax moth, *Galleria mellonella* (*IMPI*; AY330624.1) as a control, respectively. After seven days, guts were dissected in RNA*later* solution for subsequent gene expression analysis (**a**–**d**) or in protein extraction buffer for enzyme activity assays (**e**–**h**), respectively. Copy number estimates of *PcGSS1*, *PcGSS2*, *PcGSS4*, and *PcGSS5* are given per 1,000 copies of mRNA for the reference gene *RPL13a*. For GSS activity assays, gut tissue homogenates were incubated with a mixture of eight different glucosinolates (GLS) at 35 °C for 2 h, and desulfo-GLS produced were quantified by LC-MS/MS via external standard curves. Bars labeled with different letters are significantly different (details of statistical analyses are summarized in Table S3). Means + SE of n = 9–11 biological replicates. I3M, indol-3-ylmethyl; 4MSOB, 4-methylsulfinylbutyl.

To determine whether *Pc*GSS1 and *Pc*GSS2 are responsible for GSS activity in the *P. chrysocephala* gut, we silenced *PcGSS1* and *PcGSS2* expression by RNA interference. In initial experiments, the injection of dsRNAs targeting the coding sequence of *PcGSS1* or *PcGSS2* led to the non-specific knock-down of all *PcGSS* genes (data not shown). By injecting adults with dsRNAs targeting the 3′-UTR of *PcGSS1* or *PcGSS2*, we were able to specifically knock-down *PcGSS1* and *PcGSS2* gene expression by about 90%, respectively (Fig. 4a,b). However, *PcGSS4* and *PcGSS5* transcript levels were also significantly lower in both treatments compared to the control (between 43 and 80%; Fig. 4c,d). To analyze the effects of reduced *PcGSS* transcript abundance on GSS activity in *P. chrysocephala*, we incubated crude gut homogenates with a mixture of eight different glucosinolates and compared the amounts of formed desulfo-glucosinolate between treatments. In agreement with the detected *in vitro* activity, GSS activity towards sinalbin and benzyl glucosinolate was significantly lower after injection of *PcGSS1*-dsRNA than after injection of *PcGSS2-* and *IMPI-*dsRNAs, respectively (One-way ANOVA; *P* < 0.001; Fig. 4e,f). This result confirmed that *PcGSS1* encodes the sinalbin-specific GSS activity observed in crude gut homogenates of *P. chrysocephala*. Furthermore, significantly less desulfo-indol-3-ylmethyl glucosinolate was formed in assays with gut homogenates from *PcGSS2*-dsRNA-injected adults compared to the other treatments, which shows that *PcGSS2* is responsible for GSS activity towards indol-3-ylmethyl glucosinolate in *P. chrysocephala* (n = 9–11, One-way ANOVA; *P* < 0.001; Fig. 4g). Surprisingly, GSS activity towards the aliphatic 4-methylsulfinylbutyl glucosinolate was significantly reduced in both *PcGSS1-* and *PcGSS2*-dsRNA-injected adults compared to adults injected with *IMPI*-dsRNA, whereas GSS activities towards other glucosinolates did not differ between treatments (Fig. 4h, Supplementary Table S3).

### *PcGSS* evolved from arylsulfatase-like genes by gene duplication and neofunctionalization

**Figure 5:**
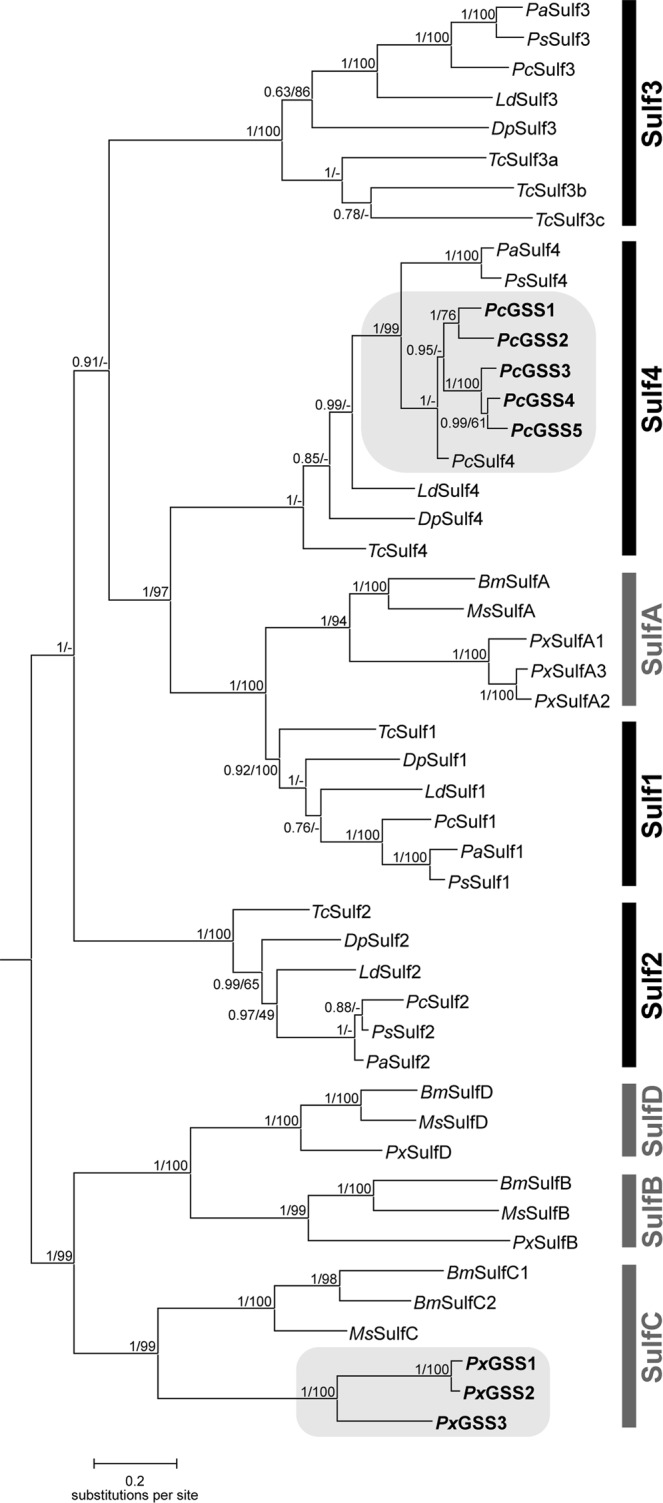
Bayesian-inferred phylogeny of coleopteran and lepidopteran arylsulfatases and GSS enzymes. Posterior probability values and bootstrap values (1,000 replicates) of a Maximum-Likelihood analysis of the same dataset are shown next to each node. Sulf1, Sulf2, Sulf3, and Sulf4 designate Coleoptera-specific clades (black vertical bars), SulfA, SulfB, SulfC, SulfD designate Lepidoptera-specific clades (grey vertical bars). Clades containing GSS enzymes are highlighted with grey background. -, not supported in Maximum-Likelihood analysis. The following species were included in the analysis: Coleoptera: *Pc*, *Psylliodes chrysocephala*; *Pa*, *Phyllotreta armoraciae*; *Ps*, *Phyllotreta striolata*; *Dp*, *Dendroctonus ponderosae*; *Ld*, *Leptinotarsa decemlineata*; *Tc*, *Tribolium castaneum*; Lepidoptera: *Bm*, *Bombyx mori*; *Ms*, *Manduca sexta*; *Px*, *Plutella xylostella*.

To investigate the evolutionary origin of GSS activity in *P. chrysocephala*, we inferred the relationships between *P. chrysocephala* and *P. xylostella* GSS and other coleopteran and lepidopteran arylsulfatase-like enzymes in Bayesian and Maximum-Likelihood analyses. We included the deduced amino acid sequences of arylsulfatase-like genes identified in the transcriptomes of *P. striolata* and *P. armoraciae* (GenBank accession numbers KX986123 to KX986130; Table 1, Supplementary Data S1), and in the genomes of the mountain pine beetle, *Dendroctonus ponderosae*, the Colorado potato beetle, *Leptinotarsa decemlineata*, and the red flour beetle, *Tribolium castaneum* (Supplementary Data S1). These coleopteran arylsulfatases clustered in four separate clades that were named Sulf1, Sulf2, Sulf3, and Sulf4. The identified *Pc*GSSs formed a subclade within the coleopteran-specific Sulf4 group, suggesting a lineage-specific diversification of *Sulf4* genes in *P. chrysocephala* (Fig. 5). While the position of *Pa*Sulf4 and *Ps*Sulf4 as a sister group to the *Pc*Sulf4/*Pc*GSS group was well supported with high posterior probability and bootstrap values (1/99), the topology within the *Pc*Sulf4/*Pc*GSS group was not unambiguously resolved (Fig. 5). However, active and weakly active *Pc*GSSs formed separate subclades in both analyses. To analyze whether different selection pressures act on these two GSS subclades, on *PcSulf4*, and on the other coleopteran *Sulf4* genes, respectively, we performed a series of branch models in codeml (Supplementary Fig. S7). Our analyses showed that similar selection pressures act on both *PcGSS* subclades (χ^2^ = 0.79, *P* = 0.37). We then tested whether different selection pressures act on the *PcGSS* clade and the ancestral *PcSulf4* gene, and again found no significant difference (χ^2^ = 3.67, *P* = 0.055). However, compared to the *Sulf4* genes from two *Phyllotreta* species (*PaSulf4*/*PsSulf4* clade, *ω* = 0.0888) and the other coleopteran *Sulf4* genes (*ω* = 0.0258), the *PcSulf4*/*PcGSS* clade is under more relaxed purifying selection (*ω* = 0.3334) (Supplementary Fig. S7).

The arylsulfatases identified in the genomes of *Bombyx mori*, *Manduca sexta*, and *P. xylostella* clustered in four lepidopteran-specific clades named SulfA, SulfB, SulfC, and SulfD. The lepidopteran SulfA clade and the coleopteran Sulf1 clade most likely share a common ancestor as suggested by high posterior probability and bootstrap values (1/100). The *P. xylostella* GSSs clustered within the lepidopteran SulfC clade separately from the *Pc*GSSs, indicating an independent evolution of GSS activity in *P. xylostella* and *P. chrysocephala* (Fig. 5).

### Metabolic fate of sinalbin in *P. chrysocephala* adults

Since crude gut homogenates from *P. chrysocephala* showed much higher GSS activity towards sinalbin than towards other tested glucosinolate substrates, we hypothesized that sinalbin is mainly detoxified by desulfation. To test this hypothesis, we fed adults with detached *Arabidopsis thaliana* leaves containing 250 nmol sinalbin. To assess whether plant myrosinase activity affects sinalbin metabolism in *P. chrysocephala*, we used leaves of *A. thaliana* Col-0 wild type plants and the myrosinase-deficient *A. thaliana tgg1* × *tgg2* double knock-out mutant. In this quantitative feeding experiment, desulfo-sinalbin accounted for 25% and 83% of the total ingested sinalbin from wild type and *tgg1* × *tgg2* leaves, respectively (Table 3). Desulfo-sinalbin was almost exclusively detected in faeces together with minor amounts of intact sinalbin, corresponding to 0.9% and 3% of the total ingested glucosinolates, respectively (Table 3). Only traces of intact and desulfo-sinalbin were present in body extracts of *P. chrysocephala*. In total, we recovered 26% of the total ingested sinalbin from wild type leaves, and 86% from the myrosinase-deficient *tgg1* × *tgg2* mutant as intact or desulfo-sinalbin.

**Table 3:** Metabolic fate of ingested sinalbin in *Psylliodes chrysocephala* adults fed on *Arabidopsis thaliana* Col-0 wild type and *tgg1* × *tgg2* double knock-out mutant leaves spiked with 250 nmol sinalbin.

Compound	Mean percentage ± SD (n = 8)^a^			
	*A. thaliana* Col-0		*A. thaliana tgg1* × *tgg2*	
	Beetle	Faeces	Beetle	Faeces
Sinalbin	0.03 ± 0.07	0.89 ± 1.00	0.05 ± 0.07	2.63 ± 1.97
Desulfo-sinalbin	0.09 ± 0.06	25.22 ± 4.04	0.30 ± 0.14	82.83 ± 16.71
Total	26.23 ± 3.82		85.82 ± 17.56	

^a^The detected amounts of intact and desulfo-sinalbin were calculated relative to the total amount of ingested sinalbin, which was set as 100%.

## Discussion

Glucosinolate desulfation catalyzed by sulfatase enzymes is a well-known detoxification strategy that prevents the activation of ingested glucosinolates to toxic isothiocyanates^16–19^. Previously, we detected desulfo-4-methylsulfinylbutyl glucosinolate in the body of *P. chrysocephala*, which implied that adults possess GSS activity^15^. In this study, we demonstrated the presence of GSS activity in the gut of *P. chrysocephala* adults, and identified five genes encoding sulfatases with distinct activities towards glucosinolates.

In contrast to diamondback moth larvae, which secrete GSS enzymes into the gut lumen^16,17^, GSS activity in *P. chrysocephala* is associated with the gut membrane. In agreement with this localization *in vivo*, all *PcGSS* genes are specifically expressed in the gut and encode enzymes with an N-terminal secretion signal and a C-terminal transmembrane domain, predicting an extracellular localization of the catalytic site in the gut lumen. While GSS activity in *P. xylostella* larvae and desert locusts exhibit broad substrate specificity^16–18^, enzyme assays with *P. chrysocephala* gut tissue homogenates revealed specific GSS activity towards sinalbin, an uncommon benzenic glucosinolate that occurs in a few Brassicaceae species, including *Sinapis alba* and *Sinapis arvensis*^25,26^. We thus expected sinalbin detoxification to be much more efficient compared to other glucosinolates in *P. chrysocephala*. Indeed, adults desulfated e.g. three times as much sinalbin as the aliphatic 4-methylsulfinylbutyl glucosinolate in quantitative feeding experiments^15^. However, in the absence of myrosinase activity, adults excreted more than 80% of the ingested sinalbin as desulfo-sinalbin (Table 3). Together, these findings indicate that a fraction of ingested sinalbin is activated by the plant myrosinase despite comparatively high GSS activity towards this substrate in the gut of *P. chrysocephala*.

Considering the broad food plant spectrum of *P. chrysocephala*^14,27^, a sinalbin-specific detoxification mechanism is surprising. Although several *Psylliodes* species including *P. chrysocephala* were reported to feed on sinalbin-containing plants such as *S. alba* and *S. arvensis*^27,28^, *P. chrysocephala* did not prefer *S. alba* and even avoided *S. arvensis* when offered the choice between leaf discs of *Sinapis* spp. and oilseed rape^14^. Moreover, our own attempts to establish a laboratory culture of *P. chrysocephala* on *S. alba* plants were unsuccessful, most likely because *S. alba* is not a suitable host plant for larval development. Thus, the ecological relevance of GSS activity towards sinalbin is currently unclear.

Most food plants of *P. chrysocephala* contain complex mixtures of aliphatic, benzenic, and indolic glucosinolates. Our previous and current results suggest that the metabolic fate of specific glucosinolate types can differ strongly^15^. For example, adults sequestered the aliphatic 4-methylsulfinylbutyl glucosinolate but not the benzenic sinalbin. In addition, desulfo-4-methylsulfinylbutyl glucosinolate was primarily detected in the body, whereas desulfo-sinalbin was excreted with the faeces^15^. Whether *P. chrysocephala* sequesters aliphatic desulfo-glucosinolates, or converts aliphatic glucosinolates to desulfo-glucosinolates after sequestration, is not yet known. The importance of different glucosinolate detoxification mechanisms in *P. chrysocephala* thus likely depends on the glucosinolate profile of their food plants.

GSS activity *in vivo* largely corresponded to the *in vitro* activity of two sulfatases we identified in the *P. chrysocephala* transcriptome, namely *Pc*GSS1 and *Pc*GSS2. Recombinant *Pc*GSS1 primarily used sinalbin as a substrate and showed minor activity towards the structurally similar benzyl glucosinolate, whereas indol-3-ylmethyl glucosinolate was the major substrate of *Pc*GSS2. Both enzymes were also responsible for GSS activity towards these glucosinolate substrates in the *P. chrysocephala* gut, as demonstrated by silencing *PcGSS1* and *PcGSS2* expression in adults. Compared to *Pc*GSS1 and *Pc*GSS2, enzyme activities of recombinant *Pc*GSS3, *Pc*GSS4, and *Pc*GSS5 were barely detectable under our assay conditions (Table 2). Although these three enzymes may act on other glucosinolates or sulfated substrates, the substitution of the conserved histidine residue in position 125 with an asparagine residue likely affects their catalytic activity (Fig. 2). However, exchanging the asparagine residue with the catalytically active histidine residue in *Pc*GSS3, *Pc*GSS4, and *Pc*GSS5 by site-directed mutagenesis did not enhance their activity towards the tested glucosinolate substrates (Supplementary Fig. S6), which indicates that additional substitutions influence their activity. Comprehensive sequence comparisons between *Pc*GSS1/2 and *Pc*GSS3/4/5 might reveal additional amino acid residues that mediate enzyme activity or determine substrate specificity.

Although the injection of dsRNAs targeting the 3′-UTRs of *PcGSS1* and *PcGSS2* led to the specific knock-down of *PcGSS1* and *PcGSS2* transcripts, respectively, expression levels of *PcGSS4* and *PcGSS5* were significantly lower in both treatments as well, most likely due to off-target effects. In addition, we observed significantly reduced GSS activity towards 4-methylsulfinylbutyl glucosinolate in crude gut homogenates in both treatments (Fig. 4h). Since only *Pc*GSS2 showed activity towards 4-methylsulfinylbutyl glucosinolate in enzyme assays, it is tempting to speculate about another so far unidentified GSS enzyme capable of desulfating 4-methylsulfinylbutyl glucosinolate in *P. chrysocephala*. However, we cannot exclude that *Pc*GSS3, *Pc*GSS4, or *Pc*GSS5 exhibit GSS activity towards 4-methylsulfinylbutyl glucosinolate *in vivo*.

GSSs in *P. xylostella* and in *P. chrysocephala* evolved independently from each other via duplications of distinct arylsulfatase-like genes (Fig. 5). In *Plutella*, duplication of *SulfC* resulted in *GSS1/2* and *GSS3*, which had both evolved under positive selection, an unusual selection pattern that was termed “concerted neofunctionalization”^17^. A second lineage-specific duplication of *GSS1/2* then led to *GSS1* and *GSS2*. In *P. chrysocephala*, GSS clustered within the coleopteran Sulf4 clade. Since recombinant *Pc*Sulf4 was active towards the general substrate 4-nitrocatechol sulfate, but not towards glucosinolates, we hypothesize that *PcSulf4* maintains the ancestral function, whereas duplicated *Sulf4* copies acquired a novel adaptive function along with a distinct tissue-specific expression pattern (Fig. 3). Within the Sulf4 clade, *Pc*GSSs clustered in two subclades comprising *Pc*GSS1/2 and *Pc*GSS3/4/5, respectively (Fig. 5). This branching pattern and our experimental data suggest that *PcGSS1* and *PcGSS2* were selected for distinct glucosinolate substrate specificities after gene duplication. It is, however, difficult to predict why *PcGSS3*, *PcGSS4*, and *PcGSS*5 have diversified, as their functions are unknown. Nevertheless, we assume that *PcGSS3*, *PcGSS4*, and *PcGSS*5 also play an important role in *P. chrysocephala*, because (i) these genes are under purifying selection, and (ii) transcript levels of *PcGSS4* and *PcGSS5* were at least 5 times higher than those of *PcGSS1* and *PcGSS2* (Supplementary Fig. S7, Fig. 3).

Arylsulfatases were found to be conserved within Lepidoptera and Coleoptera, but underwent lineage-specific duplications (Fig. 5). According to our phylogenetic analysis, only lepidopteran *SulfA* and coleopteran *Sulf1* genes evolved from a common ancestor, and thus likely represent true orthologs. The biological functions and substrates of arylsulfatases in insects are unknown. However, there is evidence that arylsulfatase activity plays a role in molting of southern armyworm larvae, possibly by metabolizing inactive sulfate esters of the molting hormone ecdysone^29,30^. In *T. castaneum*, the arylsulfatase *Tc*Sulf3a is necessary for quinone accumulation in defensive glands, but its exact role is still unclear^31^.

The catalytic activity of eukaryotic sulfatases strictly depends on an aldehyde residue, which is generated by post-translational modification of a conserved cysteine residue to a Cα-formylglycine residue. This conserved cysteine residue is replaced by a serine residue in all members of the coleopteran Sulf2 clade, except for Sulf2 from *T. castaneum*, where the cysteine residue is substituted with a glycine residue (Supplementary Fig. S8). In addition, a substitution of the cysteine residue by a glycine residue was found in the lepidopteran SulfB clade (Supplementary Fig. S8). Serine-type sulfatases are well-known in prokaryotes, which possess specific enzymes for the conversion of the serine-residue to the active Cα-formylglycine, but so far not in eukaryotes^24,32^. Since we found a single conserved serine-type sulfatase in beetle species belonging to two different subfamilies of the Chrysomelidae (Galerucinae and Chrysomelinae) as well as in a curculionid species, serine-type sulfatases may be wide-spread at least in Coleoptera. To test this hypothesis, a more comprehensive phylogenetic analysis of arylsulfatase-like sequences from members of other families in the order Coleoptera, and other insect orders, is necessary. Based on the high sequence similarity of Sulf2 to other insect arylsulfatase-like enzymes, serine-type sulfatases probably evolved from an insect cysteine-type sulfatase ancestor.

In summary, we have shown that *P. chrysocephala* possesses several GSS enzymes with distinct substrate specificities, which play a role in the detoxification of dietary glucosinolates in adults. The identified *Pc*GSSs might play a more important role in the detoxification of benzenic and indolic glucosinolates, but further studies are necessary to test this hypothesis. GSS activity evolved independently in the cabbage stem flea beetle, *P. chrysocephala*, and the diamondback moth, *P. xylostella*, by duplications of distinct arylsulfatase-like enzymes. Two other generalist herbivores, the desert locust, *S. gregaria*, and the whitefly, *B. tabaci,* also possess GSS activity, which most likely evolved from arylsulfatase-like enzymes as well. The identification of GSS enzymes in *S. gregaria* and *B. tabaci* will provide an unprecedented opportunity to analyze the independent evolution of a glucosinolate detoxification mechanism across four different insect orders.

## Experimental Procedures

### Plants and insects

Seeds of *Brassica rapa* cv. Yu-Tsai-Sum and *Brassica juncea* cv. Bau Sin were purchased from Known-You Seed (Kaohsiung, Taiwan). *Sinapis alba* seeds were purchased from Sperli GmbH (Everswinkel, Germany). Plants were cultivated in a controlled environment chamber at 21 °C, 55% relative humidity, and a 14-h light/10-h dark period. *Arabidopsis thaliana* plants were cultivated in a controlled environment chamber at 21 °C, 55% relative humidity, and a 10-h light/14-h dark period. The following *A. thaliana* genotypes were used: *A. thaliana* Col-0 wild type, the *A. thaliana tgg1* × *tgg2* double knock-out mutant lacking myrosinase activity in leaves^33^, and the *A. thaliana myb28* × *myb29* double knock-out mutant, which is devoid of aliphatic glucosinolates^34^. *Psylliodes chrysocephala* and *Phyllotreta armoraciae* flea beetles were reared on potted *B. rapa* and *B. juncea* plants, respectively, in a controlled environment chamber at 24 °C, 75% relative humidity, and a 16-h light/8-h dark period. Adults were provided with three-week old plants every week. *B. rapa* plants with *P. chrysocephala* eggs and *B. juncea* plants with *P. armoraciae* eggs were kept in separate cages for larval development for four weeks and for three weeks, respectively. Afterwards, remaining plant material was removed and the soil with pupae was kept in plastic containers (9 L volume, Lock&Lock, Seoul, Korea) until adults emerged.

### Chemicals

Sinalbin (*p*-hydroxybenzyl glucosinolate) and 4-methylthiobutyl glucosinolate were isolated from seeds of *S. alba* and *Eruca sativa*, respectively, according to Thies^35^. All other glucosinolates were purchased from Phytoplan (Heidelberg, Germany). To obtain standards of the corresponding desulfo-glucosinolates, glucosinolates were incubated with *Helix pomatia* sulfatase solution prepared according to Graser *et al*.^36^.

### Protein extraction from *P. chrysocephala* and *P. armoraciae* adults

To determine the localization of GSS activity in *P. chrysocephala*, adults were dissected into gut and remaining body tissues in chilled 20 mM 2-(*N*-morpholino)ethanesulfonic acid (MES) buffer (pH 5.2). Tissues from ten individuals were pooled per replicate and four biological replicates were prepared. Gut and remaining body tissue samples were homogenized in 200 µL and 500 µL 20 mM MES buffer (pH 5.2) supplemented with proteinase inhibitors (cOmplete™ EDTA-free, Roche, Basel, Switzerland), respectively. For comparison, gut tissue homogenates were prepared from *P. armoraciae* adults as described above. To determine whether GSS activity is soluble or associated with the gut cell membrane, half of the crude homogenate was centrifuged at 21,130 × *g* for 20 min at 4 °C. The supernatant containing soluble proteins was transferred to a new tube, and the tissue pellet was resuspended in 100 µL protein extraction buffer. The protein concentration of each sample was determined using the Bradford protein assay (Bio-Rad Laboratories, Hercules, CA, USA) according to the manufacturer’s instructions, and GSS activity assays were performed with 40 µL of each extract.

To compare the GSS activity in adults that were injected with double-stranded RNA (dsRNA) targeting *PcGSS1*, *PcGSS2*, or the *Galleria mellonella* inducible metalloproteinase inhibitor (*GmIMPI*) as a negative control (see section RNAi), dissected guts of five adults were pooled for one sample and homogenized in 100 µL 20 mM MES buffer (pH 5.2) supplemented with proteinase inhibitors (cOmplete™ EDTA-free, Roche). The protein concentration of the crude gut tissue homogenates was determined using the Bradford protein assay (Bio-Rad Laboratories), and the protein concentration was adjusted to 10 µg total protein in 50 µL 20 mM MES buffer (pH 5.2) for GSS activity assays (see below).

### Identification of arylsulfatase-like genes in the transcriptomes of *P. chrysocephala, P. armoraciae*, and *P. striolata*

Arylsulfatase-like genes were identified in the local transcriptome databases of *P. chrysocephala*, *P. armoraciae*, and *P. striolata* by tBLASTn searches using the *Plutella xylostella* GSS amino acid sequence (*Px*GSS1, CAD33828.1) as a query. Full-length sequences of open reading frames (ORFs) were obtained by rapid amplification of cDNA ends-PCR (RACE-PCR). RNA isolation and cDNA synthesis for PCR and RACE-PCR were performed as described in Beran *et al*.^22^. PCR-products were cloned into the pCR™4-TOPO™ vector (Thermo Fisher Scientific, Waltham, MA, USA) and Sanger-sequenced. Primer sequences are listed in Supplementary Data S2 and full-length nucleotide and amino acid sequences for all three flea beetle species are given in Supplementary Data S1. The manually curated sequences have been deposited in the GenBank database under accession numbers KX986114–KX986130.

### Analysis of the predicted amino acid sequences

The molecular masses and isoelectric points of the putative *P. chrysocephala* arylsulfatases were computed using the ProtParam tool^37^. The presence of a signal peptide and transmembrane domain was predicted using SignalP 4.1^38^ and Phobius^39^, respectively. Sulfatase signature motifs were predicted by PROSITE^40^. The number of putative *N*-glycosylation sites was determined using NetNGlyc 1.0 provided at DTU Bioinformatics (http://www.cbs.dtu.dk/services/NetNGlyc/).

### Cloning and expression of arylsulfatase-like genes from *P. chrysocephala*

The arylsulfatase-like genes from *P. chrysocephala* were amplified from cDNAs by PCR (Advantage 2 Polymerase Mix, Takara Bio USA, Mountain View, CA, USA; Phusion™ High-Fidelity DNA Polymerase, Thermo Fisher Scientific) and cloned into the pIB/V5-His TOPO™ TA expression vector (Thermo Fisher Scientific) or the pIEx™-4 expression vector (Merck Millipore, Darmstadt, Germany) using gene-specific primers (Supplementary Data S2). The forward primers were designed to include a 5′-Kozak translation initiation sequence and the reverse primers were designed to lack the stop codon for fusion with the vector-encoded V5 epitope or S-tag combined with a 6 × His-tag or 8 × His-tag, respectively. Arylsulfatases with a predicted C-terminal transmembrane domain were amplified without transmembrane domain. Expression constructs confirmed by Sanger-sequencing were used to transiently transfect *Sf*9 or High Five^TM^ insect cells (both from Thermo Fisher Scientific) as described in Beran *et al*.^22^. A pIB/V5-His TOPO™ TA vector containing *P. xylostella GSS1* (kindly provided by Roy Kirsch, MPI-CE) was expressed as a positive control, and insect cells treated with transfection reagent only served as a negative control. After incubation at 27 °C for 72 h, the cell culture medium containing the secreted recombinant proteins was harvested for Western blotting and activity assays. Because of low protein expression levels, recombinant *Pc*Sulf1, *Pc*Sulf2, and *Pc*GSS1 were concentrated by precipitation with 10% (v/v) trichloroacetic acid (TCA) and 0.02% deoxycholate before Western blot analysis. Recombinant proteins were detected with a His-tag antibody HRP-conjugate (dilution 1:10,000; Merck Millipore).

### Site-directed mutagenesis

For site-directed mutagenesis of the amino acid in position 125 in *Pc*GSS2, *Pc*GSS3, *Pc*GSS4, and *Pc*GSS5, specific primers possessing the mutated codon were designed according to Ho *et al*.^41^ (Supplementary Data S2). pIEx™-4 vector constructs containing the ORFs of each *PcGSS* gene without the predicted C-terminal transmembrane domain were used as templates for PCR using the Phusion™ High-Fidelity DNA Polymerase. Plasmids isolated from single colonies of transformed *E. coli* TOP10 One Shot cells (Thermo Fisher Scientific) were Sanger-sequenced, and plasmids containing the desired mutation were used to transfect High Five^TM^ insect cells as described above.

### Glucosinolate sulfatase activity assay and LC-MS/MS analysis

Assays consisted of 50 µL crude beetle protein extracts or recombinant protein and 50 µL glucosinolate solution containing a mixture of 2-hydroxy-3-butenyl-, 3-butenyl-, 4-methylsulfinylbutyl-, 4-methylthiobutyl-, indol-3-ylmethyl-, benzyl-, 2-phenylethyl- glucosinolate, and sinalbin, each at 2 mM, in 20 mM MES buffer (pH 5.2). After incubation at 35 °C for 2 h, assays were stopped by adding 200 µL of pure methanol and 200 µL of 10% (w/v) aqueous DEAE-Sephadex A-25 (GE Healthcare Life Science, Pittsburgh, PA, USA) suspension to remove remaining glucosinolates. Samples were filtrated through a syringe filter (0.2 μm, Pall Corporation, Dreieich, Germany) into glass vials and stored at −20 °C until analysis by liquid chromatography-tandem mass spectrometry (LC-MS/MS). Assays performed with boiled protein samples served as a background control. Desulfo-glucosinolates were quantified by LC-MS/MS using an Agilent 1200 HPLC system (Agilent, Santa Clara, CA, USA) connected to an API3200 tandem mass spectrometer (AB Sciex Germany GmbH, Darmstadt, Germany) as described in Beran *et al*.^15^. Multiple-reaction monitoring (MRM) was used to monitor analyte parent ion-to-product ion formation (Table S4). Data analysis was performed using Analyst Software 1.6 Build 3773 (AB Sciex). Desulfo-glucosinolates were quantified via external calibration curves.

### Arylsulfatase activity assay

Fifty microliters of culture medium from transiently transfected *Sf*9 cells were incubated with 2 mM 4-nitrocatechol sulfate (Sigma-Aldrich, St. Louis, MO, USA) in 50 mM MES buffer with 250 mM NaCl (pH 5.5) at 35 °C for 1 h. Assays were performed in triplicates together with two background controls which had been boiled at 95 °C for 5 min. After incubation, 100 µL of 0.2 M NaOH were added. The formation of 4-nitrocatechol (Sigma-Aldrich) was measured at 515 nm in a Tecan Infinite 200 spectrophotometer (Männedorf, Switzerland). The mean of the background controls was subtracted from each value, and the amounts of 4-nitrocatechol released were calculated using an external calibration curve.

### Analysis of *PcSulf* and *PcGSS* gene expression in the gut and remaining body tissues

To determine whether *PcSulf* and *PcGSS* genes are expressed in the gut and in the body (without gut), and to assess whether the glucosinolate profile of the host plant influences GSS gene expression in adults, we randomly assigned newly emerged beetles to three groups. One group was immediately dissected into gut and remaining body tissues. For each sample, five guts or bodies without gut were pooled. Dissected tissues were homogenized in 450 µL lysis buffer using three metal balls (3.5 mm diameter; Askubal, Korntal-Münchingen, Germany) in a TissueLyser (Qiagen, Hilden, Germany) at 50 Hz for 10 min. The homogenized samples were immediately frozen in liquid N_2_ and stored at −80 °C until RNA extraction. The other two groups were transferred to potted four week-old *S. alba* plants and *B. rapa* plants, respectively. After seven days feeding, adults were dissected as described above. Total RNA extraction, DNA digestion, RNA clean-up, cDNA synthesis, and quantitative real-time PCR (qRT-PCR) were performed as described in Beran *et al*.^42^. Gene-specific primers were designed manually (Supplementary Data S2), and amplification specificity was confirmed by melting curve analysis and by cloning of PCR products into the pCR™4-TOPO™ vector and Sanger-sequencing of at least 10 clones. Due to high sequence similarity between several *PcGSS* genes, it was not possible to design *PcGSS3*-specific primers. Primer efficiencies were calculated from a cDNA template dilution series. Ribosomal protein L13a (*RPL13a*) and elongation factor 1-alpha (*EF1α*) were used as reference genes. We analyzed four to five biological replicates per group, each with two technical replicates.

### Knock-down of *PcGSS* gene expression in adults by RNA interference

Double-stranded RNA (dsRNA) of an 113-bp *PcGSS1* 3′-UTR fragment, a 86-bp *PcGSS2* 3′-UTR fragment, and a 310-bp fragment of the inducible metalloproteinase inhibitor from the greater wax moth, *Galleria mellonella* (*IMPI*, AY330624.1) were synthesized using the MEGAscript™ RNAi Kit (Thermo Fisher Scientific) according to the manufacturer’s instructions. The plasmid containing the *IMPI* target sequence and primers for dsRNA synthesis were provided by Andreas Vilcinskas (University of Giessen). No potential off-targets were detected when all possible 21-mers of both RNA strands were searched against the local *P. chrysocephala* transcriptome database allowing for 2 mismatches. Two to three-day old adults were injected with 150 nL containing 90 ng of dsRNA using a Nanoliter 2010 Injector (World Precision Instruments, Sarasota, FL, USA) connected to a manual micromanipulator (MM33, Narishige, Tokyo, Japan). Injected adults were supplied with detached *B. rapa* leaves and moistened tissue and kept in a controlled environment chamber at 24 °C, 75% relative humidity, and a 14-h light/10-h dark period. Seven days after injection, guts were dissected in RNA*later*™ stabilization solution (Thermo Fisher Scientific) for gene expression analysis by qRT-PCR, or in 20 mM MES buffer (pH 5.2) for GSS activity assays (described above). For each replicate, four to six guts or rest bodies were pooled, and nine to eleven biological replicates per treatment were prepared. All samples were stored at −80 °C until analysis.

### Statistical analyses

The expression of *PcSulf* and *PcGSS* genes in the gut and the corresponding remaining body tissues relative to the reference gene *EF1α* were compared by paired *t*-test. *GSS* gene expression in newly emerged adults and adults fed for seven days on either *B. rapa* or *S. alba*, respectively, was compared by one-way analysis of variance (ANOVA) followed by Tukey’s HSD test, or the non-parametric Kruskal-Wallis test followed by Dunn’s multiple comparisons test. GSS activities in gut extracts prepared from dsRNA-injected adults, and *PcGSS* gene expression in gut tissue relative to the reference gene *RPL13a* were compared by ANOVA followed by Tukey’s HSD test, or by Kruskal-Wallis test followed by Dunn’s multiple comparisons test. All statistical analyses were performed in SigmaPlot 11.0.

### Phylogenetic analysis

In addition to the arylsulfatase-like genes identified in the transcriptomes of three flea beetle species, putative arylsulfatase genes were searched by tBLASTn in publicly available genome databases of three coleopteran species: *D. ponderosae* (Curculionidae)^43^, *L. decemlineata* (Chrysomelidae)^44^, and *T. castaneum* (Tenebrionidae)^45^, and three lepidopteran species: *B. mori* (Bombycidae)^46^, *M. sexta* (Sphingidae)^47^, and *P. xylostella* (Plutellidae)^48^ (Supplementary Data S1). Deduced arylsulfatase amino acid sequences were aligned using MAFFT with default settings (version 7, https://mafft.cbrc.jp/alignment/software/; L-INS-I algorithm). Poorly aligned regions were removed from the alignment using GBlocks (version 0.91b) with parameters set as follows: minimum length of a block after cleaning: 5; minimum number of sequences for a conserved position and for a flanking position: 25; maximum number of contiguous nonconserved positions: 8; allowed gap positions: half. The Maximum-Likelihood tree was reconstructed in MEGA 5.05 using the WAG + G + I model with pairwise deletion of gaps, and tested with 1,000 bootstrap replicates. A Bayesian phylogenetic analysis consisting of four Markov chains with 10,000,000 generations that were sampled every 100 generations was carried out in MrBayes v.3.2.1. The first 25% of saved trees were discarded.

### Selection analysis

A selection analysis was performed with a dataset containing all coleopteran *Sulf4* and *GSS* genes included in the phylogenetic analyses. The coding sequences were translated into protein sequences and aligned using the MUSCLE algorithm implemented in MEGA 7.0.14. A Maximum-Likelihood tree was reconstructed in MEGA 7.0.14 using the LG + G model with pairwise deletion of gaps. The codeml software implemented in the PAML package was used to analyze the selection pressure on the *P. chrysocephala GSS* and *Sulf4* genes and other coleopteran *Sulf4* genes^49^. First, we compared the one-ratio model, which allows one single *ω* value (the ratio of nonsynonymous (*d*_N_) to synonymous (*d*_S_) substitution rate) for all branches (*ω* = 0.1355) and a three-ratio model which allows different selection pressures to act on the *PcGSS* genes (assuming both clades are under the same selection pressure), on the ancestral *PcSulf4* gene, and on all other coleopteran *Sulf4* genes (Supplementary Fig. S7). A likelihood ratio test^50^ revealed that the three-ratio model (ln L = −11,842.05) was significantly better than the one ratio model (ln L = −11,937.49) (χ^2^ = 190.88, *P* < 0.001). In the second step, we compared the three-ratio model with a four-ratio model, which allows different selection pressures to act on the two separate *GSS* subclades, on the ancestral *PcSulf4* gene, and on the remaining coleopteran *Sulf4* genes, but there was no significant difference between both models (ln L = −11,841.65) (χ^2^ = 0.79, *P* = 0.37). To test whether different selection pressures act on the *PcGSS* clade and the ancestral *PcSulf4* gene, we first compared the one-ratio model (see above) with a two-ratio model that allows different selection pressures to act on the *PcSulf4*/*PcGSS* clade and the remaining coleopteran *Sulf4* genes. A likelihood ratio test showed that the two-ratio model is significantly better than the one-ratio model (ln L = −11,843.51) (χ^2^ = 187.95, *P* < 0.001). The two-ratio model was then compared with another three-ratio model, which assumes that the entire *PcGSS* clade, the ancestral *PcSulf4* gene, and the remaining coleopteran *Sulf4* genes are under different selection pressures. These two models were almost significantly different according to the result of the likelihood ratio test (ln L = −11,841.67) (χ^2^ = 3.67, *P* = 0.055). To test whether independent selection pressures act on the *PcSulf4/PcGSS* clade, the *PaSulf4/PsSulf4* clade, and on the other coleopteran *Sulf4* genes, we first compared the one-ratio model with a two ratio model (*PcGSS/PcSulf4/PaSulf4/PsSulf4*, and remaining *Sulf4* genes), which was significantly better than the one-ratio model (ln L = −11,869.69) (χ^2^ = 135.60, *P* < 0.001). Finally, we compared the two-ratio model with a three-ratio model, which allows the *PcGSS/Sulf4* clade, the *PaSulf4/PsSulf4* clade, and the remaining coleopteran *Sulf4* genes to have distinct selection pressures. According to the likelihood ratio test, the three-ratio model was significantly better than the two-ratio model (ln L = −11,835.16) (χ^2^ = 69.04, *P* < 0.001). The corresponding *ω* values of the final three-ratio model are shown in Supplementary Fig. S7.

### Metabolic fate of sinalbin in *P. chrysocephala* adults

To determine how much ingested sinalbin is detoxified by desulfation, we fed adults with detached *A. thaliana* Col-0 wild type and *tgg1* × *tgg2* double knock-out mutant leaves containing 250 nmol sinalbin prepared as described in Schramm *et al*.^51^. The feeding experiment and sample preparation was performed as described in Beran *et al*.^15^. In brief, we determined the remaining amount of intact sinalbin in each leaf after adult feeding to calculate how much sinalbin adults had ingested. We recovered 89.2% and 95.4% of the spiked sinalbin from *A. thaliana* Col-0 and *tgg1* × *tgg2* leaves without beetles, respectively, which showed that minor amounts of sinalbin were metabolized in leaves of both *A. thaliana* lines. Adults and faeces were collected after one additional day of feeding on a non-spiked *A. thaliana myb28* × *myb29* double knock-out mutant leaf to ensure that the plant material containing sinalbin had been digested and excreted. Desulfo-sinalbin present in *P. chrysocephala* body and faeces samples was quantified by LC-MS/MS as described above (section Glucosinolate sulfatase activity assay and LC-MS/MS analysis). Intact sinalbin was analyzed using the LC conditions and MS conditions described for desulfo-GLS and intact GLS, respectively, in Beran *et al*.^15^. Parameters for MRM analysis of sinalbin are shown in Table S1. Intact and desulfo-sinalbin were quantified via external calibration curves.

## Supplementary information

Supplementary Figures and Tables

Dataset S1

Dataset S2

## Data Availability

All data generated or analyzed during this study are included in the main text or supplement of this published article.
