# Supplementary material for: Identification and evolution of glucosinolate sulfatases in a specialist flea beetle
Source: Sci Rep. 2019 Oct 31;9:15725. doi: 10.1038/s41598-019-51749-x (PMC6823443; doi:10.1038/s41598-019-51749-x)
Supplement: Supplementary file 1 — Supplementary Figures and Tables [file 41598_2019_51749_MOESM1_ESM.pdf]

Supplementary Information

Identification and evolution of glucosinolate sulfatases in a specialist flea beetle

Seung-Joon Ahn, Franziska Betzin, Matilda W. Gikonyo, Zhi-Ling Yang, Tobias G. Köllner, Franziska Beran*

*Correspondence: Franziska Beran: fberan@ice.mpg.de

Supplementary Figures and Tables

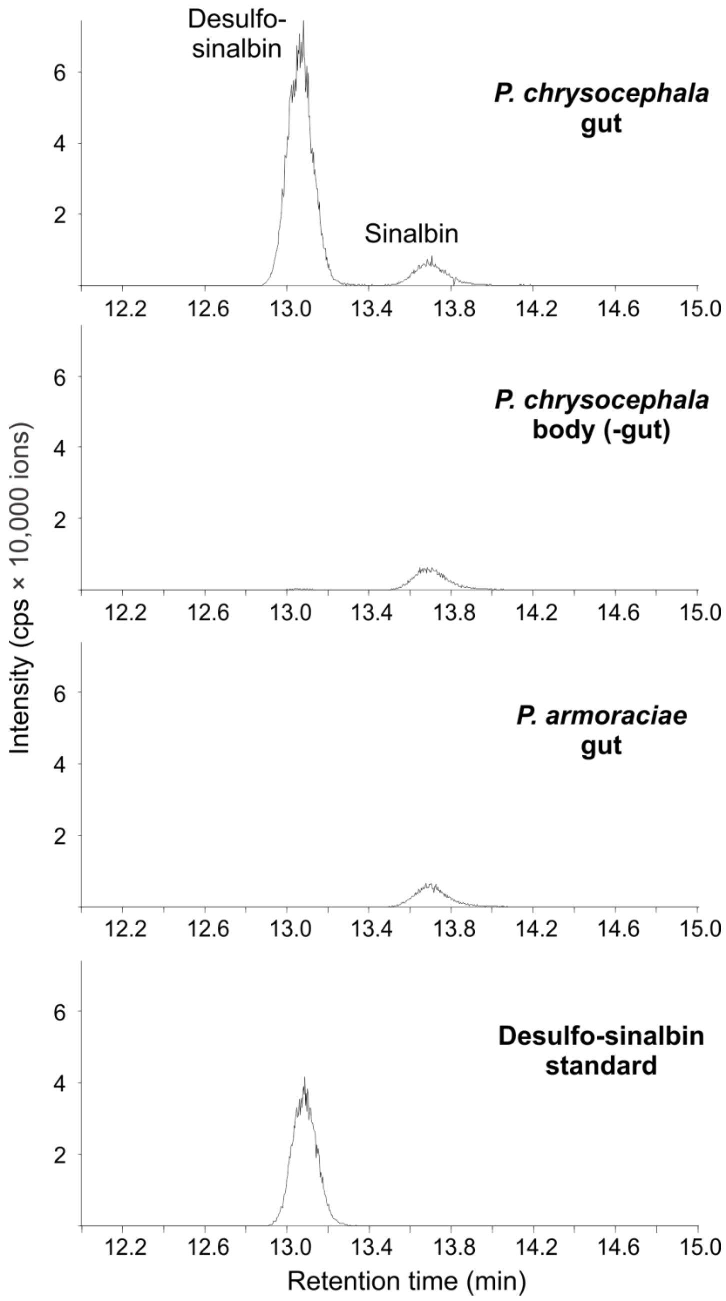

Supplementary Figure S1. Glucosinolate sulfatase activity in *Psylliodes chrysocephala* and *Phyllotreta armoraciae*. Crude protein extracts from the gut and the remaining body tissues were incubated with eight different glucosinolates. The extracted LC-MS/MS ion chromatogram of desulfo-sinalbin is shown. Remaining intact sinalbin in the assay is detected as desulfo-sinalbin due to in-source fragmentation in positive ionization mode.

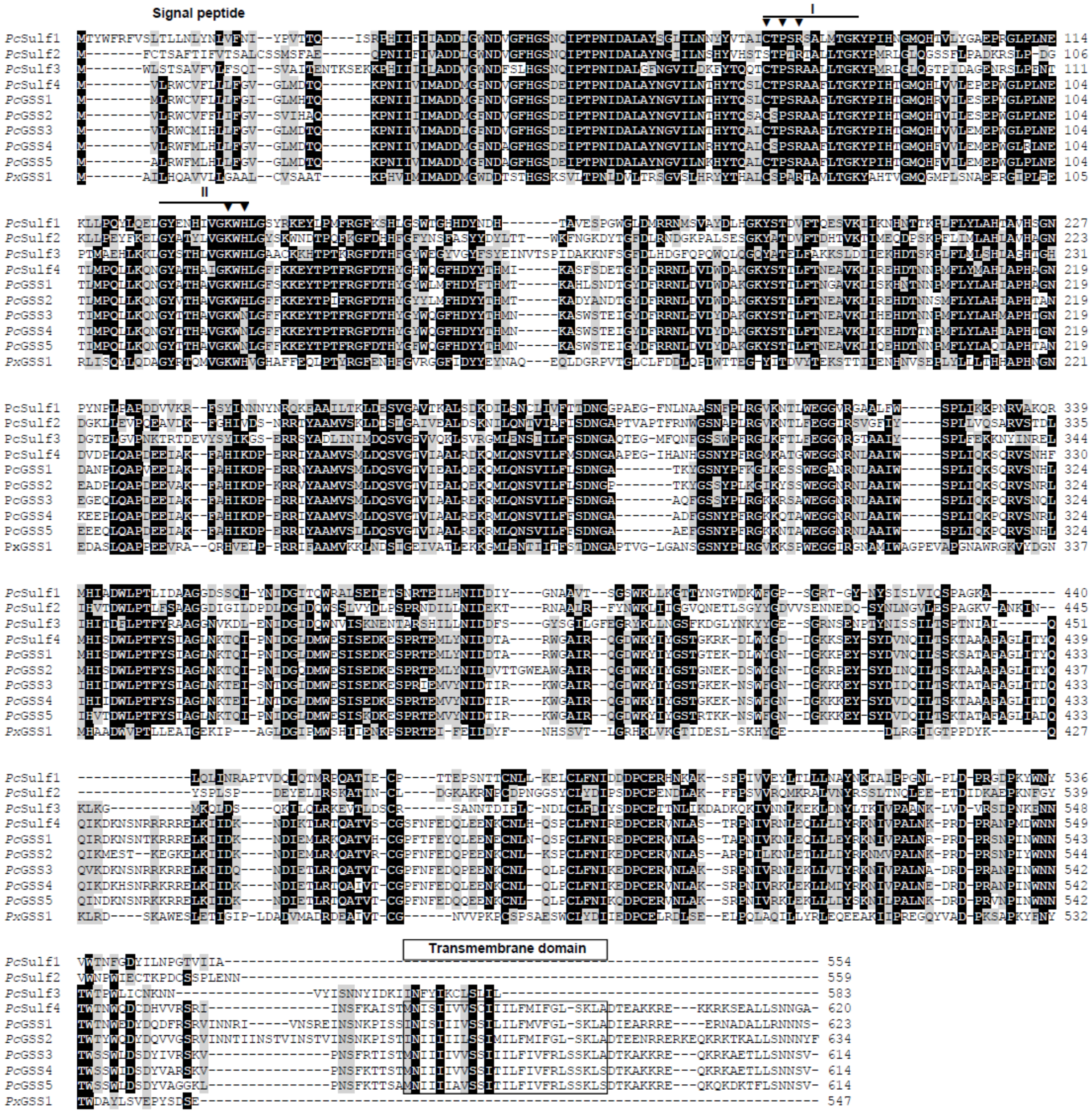

Supplementary Figure S2. Amino acid sequence alignment of arylsulfatase-like enzymes (Sulfs) and glucosinolate sulfatases (GSSs) from *Psylliodes chrysocephala* and the *Plutella xylostella* GSS1 (*Px*GSS1). Two conserved signature sequences of arylsulfatases are marked with a bar above the alignment and catalytically active residues are marked with ▼. All enzymes possess an N-terminal signal peptide. The C-terminal transmembrane domain is marked with a black frame. The threshold for similarity (grey)/identity (black) shading was set to 70%.

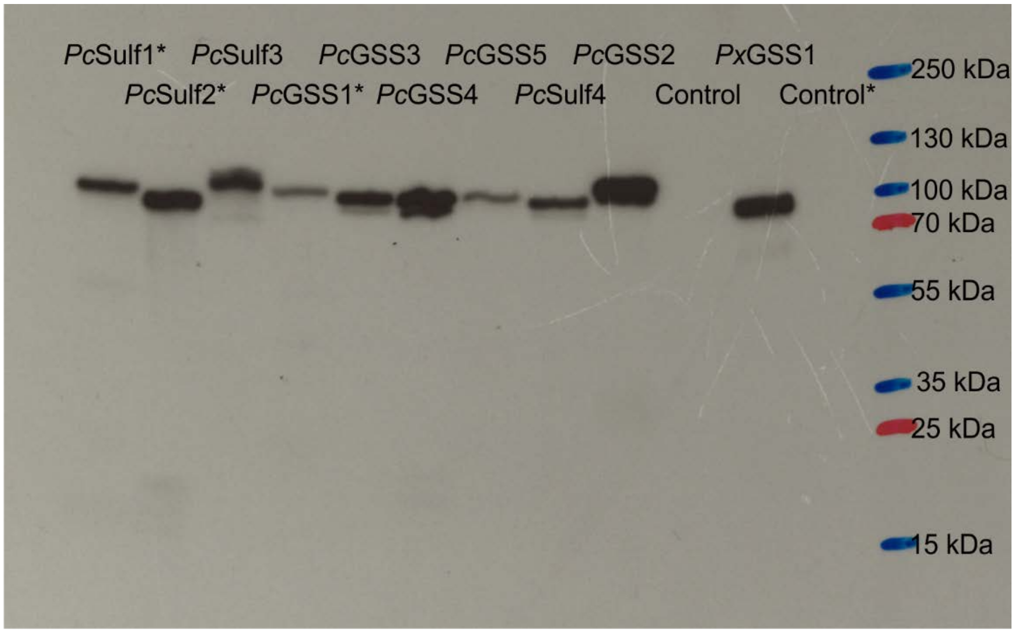

Supplementary Figure S3. Detection of recombinant *Pc*Sulfs, *Pc*GSSs, and *Px*GSS1 by Western blotting. Proteins were heterologously expressed in *Sf*9 insect cells*.* Protein samples marked with an asterisk were concentrated by trichloroacetic acid (TCA) precipitation.

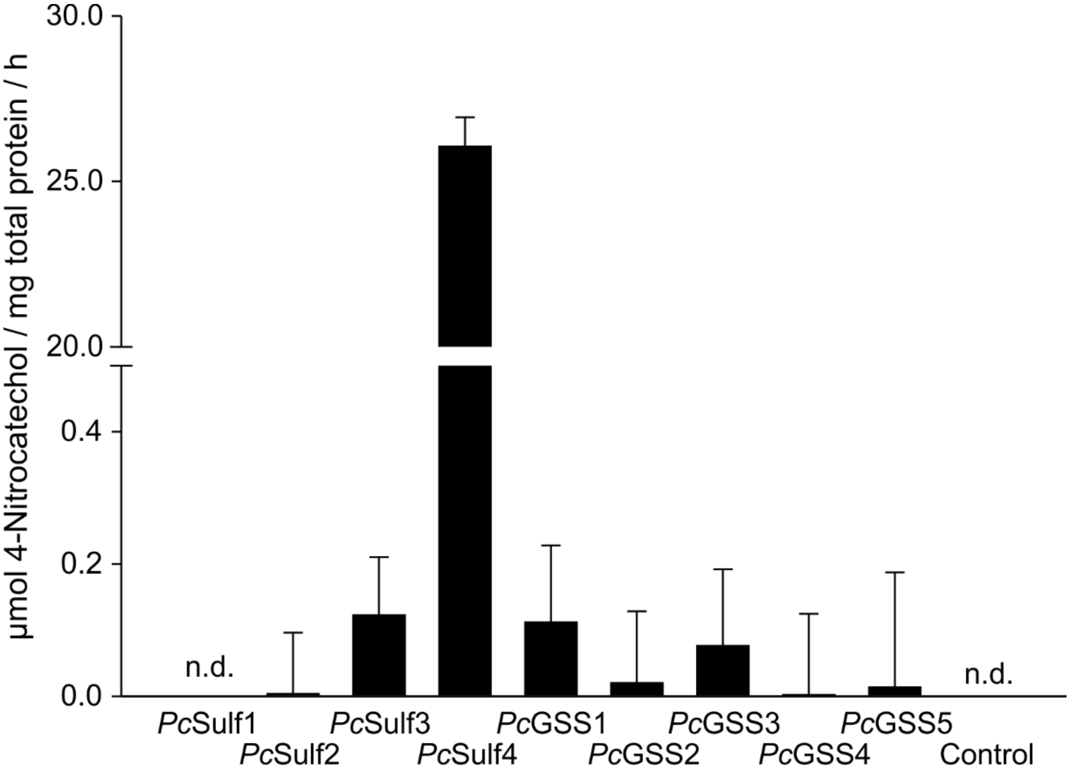

Supplementary Figure S4. Activity of recombinant sulfatases towards the general substrate 4-nitrocatechol sulfate. Proteins were heterologously expressed in *Sf*9 insect cells and non-transfected *Sf*9 cells served as a control. n.d., not detected. Means+SD of n=3 technical replicates.

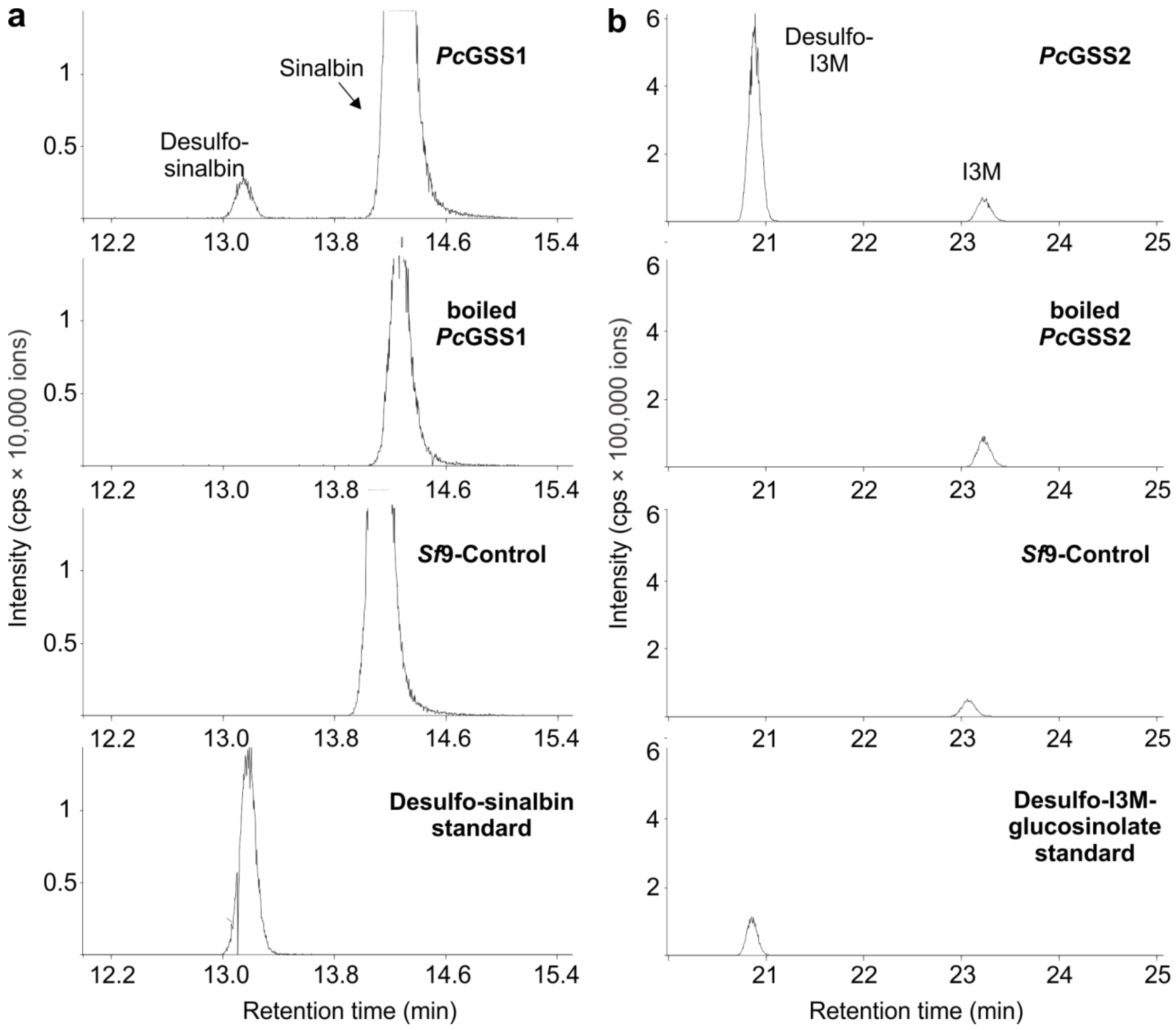

Supplementary Figure S5. LC-MS/MS analyses of glucosinolate sulfatase activity assays using *Sf*9-expressed *Pc*GSS1 (a) and *Pc*GSS2 (b). Heterologously expressed enzymes were incubated with a mixture of eight glucosinolates. Extracted LC-MS/MS ion chromatograms of desulfo-sinalbin (**a**) and desulfo-indol-3-ylmethyl glucosinolate (desulfo-I3M glucosinolate) (**b**) are shown. Remaining intact glucosinolate in the assay was detected as desulfo-glucosinolate due to in-source fragmentation in positive ionization mode.

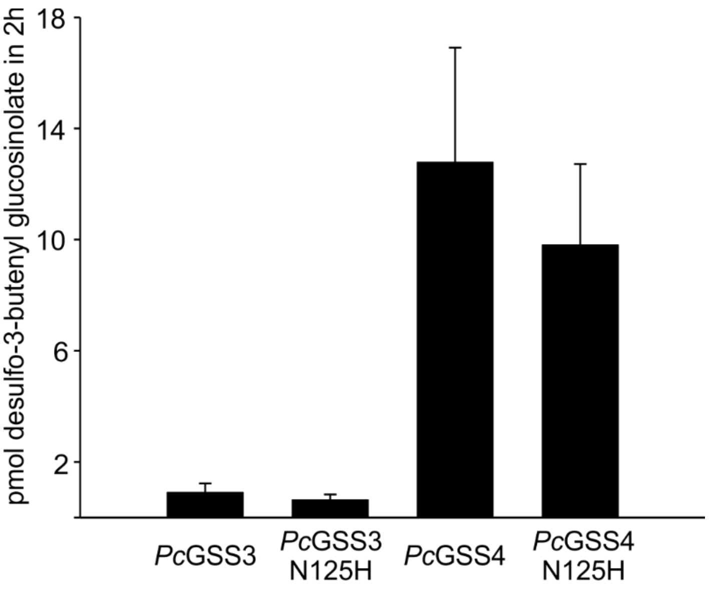

Supplementary Figure S6. Mutation of the asparagine residue at position 125 does not affect glucosinolate sulfatase (GSS) activity of *Pc*GSS3 and *Pc*GSS4 towards the substrate 3-butenyl glucosinolate. *Pc*GSS3, *Pc*GSS3 N125H, *Pc*GSS4, and *Pc*GSS4 N125H were heterologously expressed in High Five^{TM} insect cells and incubated with eight different glucosinolate substrates for 2 h at 35°C. Means+SD of n=3 technical replicates.

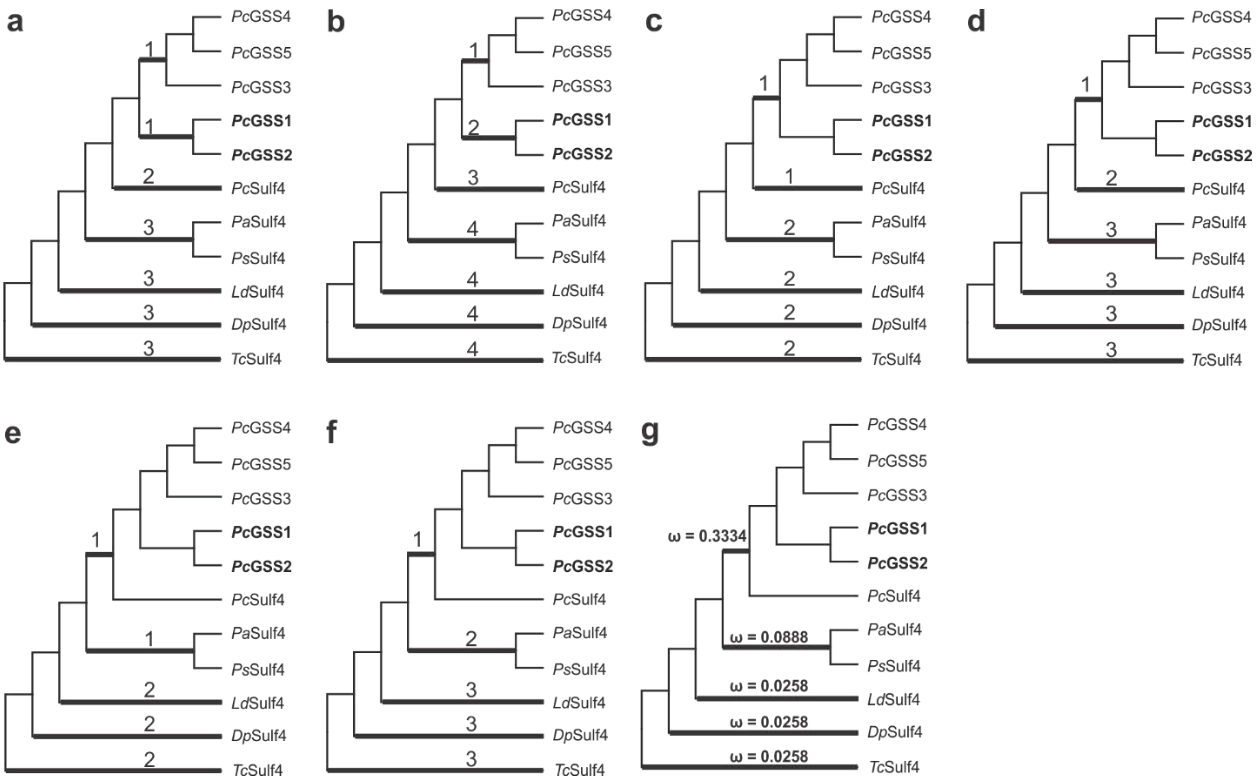

Supplementary Figure S7. Analysis of selection pressures acting on *PcGSS* and *PcSulf4* genes and other members of the coleopteran Sulf4 clade. The Maximum-Likelihood tree of coleopteran Sulf4 and GSS enzymes was constructed using MEGA 7.0.14, rooted with *Tc*Sulf4. The two major active *GSS* are shown in bold. Panels (**a**) to (**f**) show the series of models that were used to determine whether different selection pressures act on the two *PcGSS* subclades (**a** and **b**), on the entire *PcGSS* clade and the ancestral *PcSulf4* gene (**c** and **d**), and on the *PcGSS/PcSulf4* clade, the *PaSulf4/PsSulf4* clade, and other coleopteran *Sulf4* genes (**e** and **f**). The *ω* ratios calculated with the final three-ratio model are shown on the same tree in (**g**). All clades are under purifying selection. *Pc, Psylliodes chrysocephala*; *Pa*, *Phyllotreta armoraciae*; *Ps*, *Phyllotreta striolata*; *Dp*, *Dendroctonus ponderosae*; *Ld*, *Leptinotarsa decemlineata*; *Tc*, *Tribolium castaneum.*

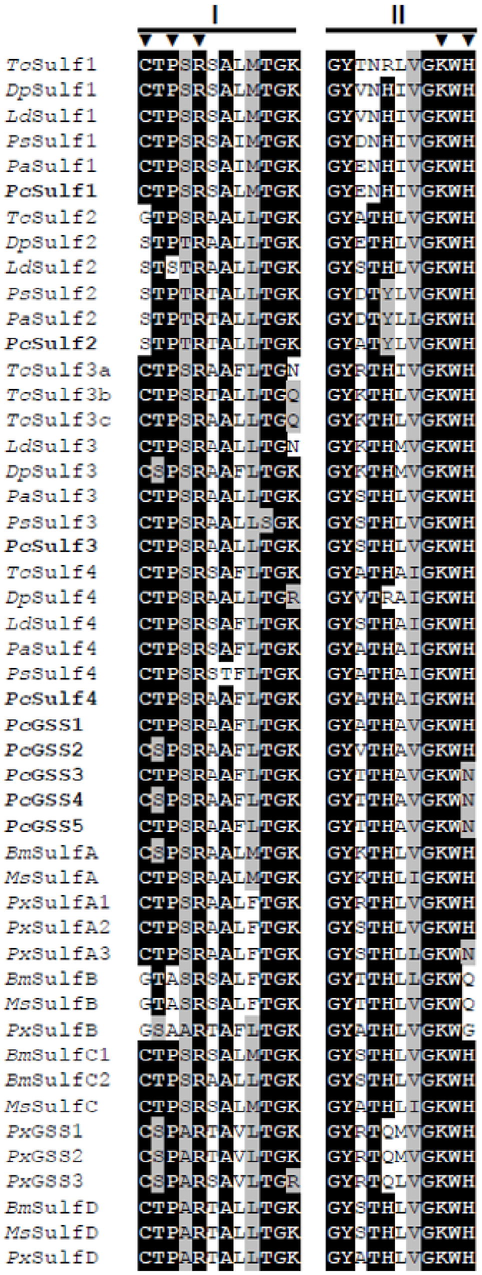

Supplementary Figure S8. Alignment of signature sequences I and II from coleopteran and lepidopteran arylsulfatase-like enzymes (Sulfs) and glucosinolate sulfatases (GSS). Catalytically active residues are marked with ▼. The threshold for similarity (grey)/identity (black) shading was set to 80%. Coleoptera: *Pc*, *Psylliodes chrysocephala*; *Pa*, *Phyllotreta armoraciae*; *Ps*, *Phyllotreta striolata*; *Dp*, *Dendroctonus ponderosae*; *Ld*, *Leptinotarsa decemlineata*; *Tc*, *Tribolium castaneum*; Lepidoptera: *Bm*, *Bombyx mori*; *Ms*, *Manduca sexta*; *Px*, *Plutella xylostella.*

Supplementary Table S1. Comparison of *PcSulf* and *PcGSS* gene expression in the gut and the rest body of *Psylliodes chrysocephala* by paired *t*-test.

Gene	**Newly emerged adults** (n=4)		**7 day-old adults fed on *Sinapis alba*** (n=5)		**7 day-old adults fed on *Brassica rapa*** (n=4)	
PcSulf1	*t*=1.346	*P*=0.271	*t*=-0.227	*P*=0.832	*t*=-6.824	*P*=0.006
PcSulf2	*t*=-5.098	*P*=0.015	*t*=-34.550	*P*<0.001	*t*=-19.129	*P*<0.001
PcSulf3	*t*=-8.736	*P*=0.003	*t*=-54.822	*P*<0.001	*t*=-11.728	*P*=0.001
PcSulf4	*t*=-10.722	*P*=0.002	*t*=-9.245	*P*<0.001	*t*=-57.978	*P*<0.001
PcGSS1	*t*=18.188	*P*<0.001	*t*=6.643	*P*=0.003	*t*=2.666	*P*=0.076
PcGSS2	*t*=2.444	*P*=0.092	*t*=2.569	*P*=0.062	*t*=3.554	*P*=0.038
PcGSS4	*t*=11.017	*P*=0.002	*t*=14.360	*P*<0.001	*t*=-4.991	*P*=0.015
PcGSS5	*t*=12.013	*P*=0.001	*t*=11.033	*P*<0.001	*t*=5.446	*P*=0.012

Supplementary Table S2. Comparison of *PcGSS* gene expression in newly emerged adults and after feeding for seven days on *Sinapis alba* and *Brassica rapa*, respectively.

Gene	Method	Statistics	*P* value
PcGSS1	Kruskal-Wallis test	*H*=8.523	*P*=0.005
PcGSS2	Kruskal-Wallis test	*H*=2.443	*P*=0.319
PcGSS4	ANOVA	*F*=4.145	*P*=0.046
PcGSS5	ANOVA	*F*=1.143	*P*=0.354

Supplementary Table S3. Comparison of *PcGSS* gene expression and GSS activity in gut tissue of adult *Psylliodes chrysocephala* injected with dsRNA targeting *PcGSS1*, *PcGSS2*, and *IMPI*, respectively.

Gene	Transformation	Method	Statistics	*P* value
PcGSS1	Log_{10}	ANOVA	*F*=75.988	*P*<0.001
PcGSS2	-	Kruskal-Wallis test	*H*=9.231	*P*=0.01
PcGSS4	-	ANOVA	*F*=12.662	*P*<0.001
PcGSS5	-	ANOVA	*F*=58.117	*P*<0.001
**Desulfo-glucosinolate**				
desulfo-3But	-	Kruskal-Wallis test	*H*=1.801	*P*=0.406
desulfo-4MSOB	$Log_{10}(x+0.01)$	ANOVA	*F*=5.065	*P*=0.014
desulfo-4MTB	$Log_{10}(x+0.01)$	ANOVA	*F*=2.486	*P*=0.102
desulfo-I3M	Log_{10}	ANOVA	*F*=10.552	*P*<0.001
desulfo-2PE	-	Kruskal-Wallis test	*H*=0.113	*P*=0.954
desulfo-Benzyl	Log_{10}	ANOVA	*F*=12.514	*P*<0.001
desulfo-Sinalbin	Log_{10}	ANOVA	*F*=21.001	*P*<0.001

3But, 3-butenyl; 4MSOB, 4-methylsulfinylbutyl; 4MTB, 4-methylthiobutyl; I3M, indol-3-ylmethyl; 2PE, 2-phenylethyl

Supplementary Table S4. LC-MS/MS parameters for multiple reaction monitoring (MRM) of desulfo-glucosinolates and sinalbin.

Compound	Q1 [*m/z*]	Q3 [*m/z*]	DP (V)	EP (V)	CE (V)	CXP (V)
desulfo-2OH3But	310.0	148.0	30	5	15	5
desulfo-3But	294.0	132.0	30	5	15	5
desulfo-4MSOB	358.0	196.0	30	5	15	5
desulfo-4MTB	342.0	180.0	30	5	15	5
desulfo-I3M	369.0	207.0	30	5	15	5
desulfo-2PE	344.1	182.1	30	5	15	5
desulfo-Benzyl	330.0	168.0	30	5	15	5
desulfo-Sinalbin	346.0	184.0	30	5	15	5
Sinalbin	424.0	95.9	-65	-5	-60	0

2OH3But, 2-hydroxy-3-butenyl; 3But, 3-butenyl; 4MSOB, 4-methylsulfinylbutyl; 4MTB, 4-methylthiobutyl; I3M, indol-3-ylmethyl; 2PE, 2-phenylethyl
